# Medication adherence and clinical outcomes in dispensing and non-dispensing practices: a cross-sectional analysis

**DOI:** 10.3399/bjgp20X713861

**Published:** 2020-12-01

**Authors:** Mayam Gomez-Cano, Bianca Wiering, Gary Abel, John L Campbell, Christopher E Clark

**Affiliations:** Primary Care Research Group, Institute of Health Services Research, University of Exeter Medical School, Exeter.; Primary Care Research Group, Institute of Health Services Research, University of Exeter Medical School, Exeter.; Primary Care Research Group, Institute of Health Services Research, University of Exeter Medical School, Exeter.; Primary Care Research Group, Institute of Health Services Research, University of Exeter Medical School, Exeter.; Primary Care Research Group, Institute of Health Services Research, University of Exeter Medical School, Exeter.

**Keywords:** diabetes mellitus, hypertension, medication adherence, primary health care, quality indicators, therapeutic adherence and compliance

## Abstract

**Background:**

Most patients obtain medications from pharmacies by prescription, but rural general practices can dispense medications. The clinical implications of this difference in drug delivery are unknown. This study hypothesised that dispensing status may be associated with better medication adherence. This could impact intermediate clinical outcomes dependent on medication adherence in, for example, hypertension or diabetes.

**Aim:**

To investigate whether dispensing status is associated with differences in achievement of Quality and Outcomes Framework (QOF) indicators that rely on medication adherence.

**Design and setting:**

Cross-sectional analysis of QOF data for 7392 general practices in England.

**Method:**

QOF data from 1 April 2016 to 31 March 2017 linked to dispensing status for general practices with list sizes ≥1000 in England were analysed. QOF indicators were categorised according to whether their achievement depended on a record of prescribing only, medication adherence, or neither. Differences were estimated between dispensing and non-dispensing practices using mixed-effects logistic regression, adjusting for practice population age, sex, deprivation, list size, single-handed status, and rurality.

**Results:**

Data existed for 7392 practices; 1014 (13.7%) could dispense. Achievement was better in dispensing practices than in non-dispensing practices for seven of nine QOF indicators dependent on adherence, including blood pressure targets. Only one of ten indicators dependent on prescribing but not adherence displayed better achievement; indicators unrelated to prescribing showed a trend towards higher achievement by dispensing practices.

**Conclusion:**

Dispensing practices may achieve better clinical outcomes than prescribing practices. Further work is required to explore underlying mechanisms for these observations and to directly study medication adherence rates.

## INTRODUCTION

In some countries medications are both dispensed in pharmacies and issued directly to patients at primary care sites. UK general practices can hold contractual rights to dispense medication to patients who live >1 mile (1.6 km) from the nearest registered pharmacy.^1^^–^^3^ Such dispensing practices are predominantly rural, where geographical barriers to alternative sources of medication and health care coexist.^4^ Both rurality and GPs’ dispensing of medications may affect quality of care and health outcomes.^5^ Demographically, rural populations have slightly higher life expectancy, with higher proportions of older people in comparison with urban areas.^6^^,^^7^ Dispensing practices are less likely to be single-handed,^8^ and have shorter opening times than pharmacies. Historically, trained dispensers have run primary care dispensaries; however, pharmacists are increasingly becoming integrated members of the primary healthcare team in all types of practices.^9^^,^^10^ In dispensing practice patient records, allergies and comorbidities are fully accessible to pharmacists and dispensers.^10^ Importantly, patients of dispensing practices can leave in possession of their prescribed medication, whereas in prescribing practices they leave with a prescription for dispensing elsewhere by a registered pharmacy. This raises the hypothesis that adherence to prescribed medications may be greater for patients of dispensing practices than for non-dispensing practices, by virtue of streamlined access to medications.

Non-adherence to prescription medication is a major cause of non-response to treatment. Between 11% and 19% of prescriptions are not actually dispensed to the patient, and barriers to medication possession exist at patient, doctor, and healthcare system levels.^11^ Easy access to on-site pharmacy services may improve medication uptake and adherence,^12^^,^^13^ overcoming logistical barriers that keep patients from presenting their prescriptions elsewhere.^14^ These barriers are reduced or absent when patients attend dispensing practices. Patients’ medication beliefs,^15^ and concerns about taking medication,^16^^–^^18^ also play a role in medication adherence. A collaborative patient–physician relationship may be key to achieving positive beliefs about treatment and increasing adherence.^19^^,^^20^ Incorporating the act or discussion of dispensing into consultations may modify patients’ beliefs, since patients report higher levels of trust in their GPs than in community pharmacists.^21^ Furthermore, GPs are more likely to be aware of patients’ personal and medical circumstances than pharmacists; therefore, they may better tailor their information to patients’ needs, taking account of issues such as health literacy.^22^^,^^23^

Reduced logistical barriers, opportunities to address patients’ beliefs, and tailoring of information to the patients’ needs may thus all influence medication adherence. However, to the authors’ knowledge, no research has yet investigated how the dispensing status of practices may impact clinical outcomes dependent on good medication adherence. This study hypothesised that on-site dispensing of medication may overcome some barriers to medication possession in comparison with the giving of a prescription. Medication adherence is not systematically recorded in primary care, but NHS Quality and Outcomes Framework (QOF) indicators are. QOF indicators include some measures of intermediate outcomes whose achievement is dependent on medication adherence, others where achievement reflects prescribing irrespective of adherence, and a third group where achievement is unrelated to prescribing. Therefore, this study investigated how dispensing practices differ from non-dispensing practices in demographic profile and sought to establish whether dispensing status is independently associated with better clinical outcomes, defined as higher achievement of QOF indicators that depend on medication adherence, than other groups of indicators.

**Table:** How this fits in

Around 15% of prescriptions given out by GPs do not get dispensed by pharmacies. In dispensing general practices, medications are usually dispensed, as opposed to prescriptions being issued to patients. This study hypothesised that this organisational difference may promote greater medication adherence for patients of dispensing practices by streamlining the issuing of medications. Quality and Outcomes Framework (QOF) indicators were studied and higher achievement levels of blood pressure and other targets were found for dispensing than for non-dispensing practices. Dispensing practices show greater achievement of QOF targets dependent on medication adherence than do non-dispensing practices. Further study is required to establish the mechanisms contributing to these findings.

## METHOD

### Study design and setting

Cross-sectional analyses were undertaken of QOF clinical indicator data from 1 April 2016 to 31 March 2017, obtained from NHS Digital^24^ and linked to dispensing practice data from March 2017, obtained from the NHS Business Services Authority.^25^ Data from March 2017 on practice population age, sex, list size, practice deprivation score, and workforce were also obtained from NHS Digital.^24^ Practices were classified as rural or urban using Office for National Statistics classification based on postcodes.^26^ Datasets were linked to QOF and dispensing status using practice codes. All data are in the public domain; thus, no ethical approval was required.

### Outcome measures

QOF performance indicators were classified into three groups according to their relation to prescribing: Group 1 was dependent on medication adherence, requiring the taking of a medication (for example, indicators reporting percentages of patients meeting pre-specified blood pressure targets); Group 2 was achieved by evidence of prescription of a medication regardless of adherence (for example, indicators reporting percentage of patients with coronary heart disease with a record of antiplatelet or anticoagulant prescribing within the preceding year). The remaining QOF indicators were unrelated to specific medications (Group 3); for example, the percentage of patients with stroke referred for further investigation. Group classification of indicators was achieved through consensus by discussion between three authors.

Given organisational differences in the processes of obtaining medication between dispensing and non-dispensing practices, and the hypothesis that these differences may affect medication adherence, a greater achievement of indicators by dispensing practices compared with non-dispensing practices was expected in Group 1, while indicators from Group 2 should show no consistent differences. Thus Group 2 represented a control set of indicators subject to any underlying trends according to dispensing status except differences in medication adherence. Group 3 provided further information on any underlying trends.

### Statistical analysis

The raw counts of eligible patients (that is, all patients fitting the corresponding indicator criterion, including those reported as exceptions) and of patients achieving each indicator in the QOF data were used. QOF business rules allow doctors to report as exceptions certain patients from any indicator so that practices are not penalised financially for inappropriate reasons. Raw figures include any patients subsequently excluded through the exception reporting process. Thus raw data overcome any risk of bias due to variation in rates of exception reporting between practices. Similarly, missing data were not an issue since all people on a disease register were included in the denominator whether or not they had the appropriate outcome recorded.

For each indicator unadjusted and adjusted mixed-effects grouped logistic regression models were fitted, with numbers of patients at each practice achieving the indicator as numerator and the number of eligible patients at each practice as denominator. Type of practice (dispensing or not dispensing) was included as a fixed effect with practice as random effects. Adjusted models included the following practice-level population characteristics: percentage of practice population aged ≥65 years, sex distribution, practice deprivation score, list size, single-handed status, and rurality.^27^ Analyses were restricted to practices with list sizes ≥1000.

## RESULTS

Data existed for 7392 practices and 1014 (13.7%) had dispensing status. Dispensing practices had more patients aged ≥65 years, fewer deprived patients, were less often single-handed, were more often rurally located, and had slightly larger list sizes when compared with non-dispensing practices (Table 1).

**Table 1.** Characteristics of dispensing and non-dispensing practices in England

	**Dispensing, *N*= 1014**	**Not dispensing, *N*= 6378**	**Total, *N*= 7392**
**Age >65 years, median, % (IQR)**	23.7 (20.9–26.9)	16.2 (11.3–20.3)	17.3 (12.2–21.6)
**Male, median, % (IQR)**	49.4 (48.8–50.0)	49.8 (48.9–51.1)	49.7 (48.9–50.9)
**Single-handed practices, *n* (%)**	28 (2.8)	476 (7.5)	504 (6.8)
**IMD least deprived, *n* (%)**	424 (41.8)	1004 (15.7)	1420 (19.2)
**Rural, *n* (%)**	717 (70.7)	375 (5.9)	1092 (14.8)
**List size, median, *n* (IQR)**	7016 (4538–10 558)	6795 (4200–10 096)	6825 (4245–10 169)

IMD = Index of Multiple Deprivation. IQR = interquartile range.

### Group 1: prescribing indicators dependent on adherence

In adjusted and unadjusted models, the odds ratio (OR) for association with dispensing status was >1 for all nine indicators, indicating higher achievement in dispensing practices. In unadjusted analyses, this only failed to reach significance for one indicator: percentage of patients with diabetes having total cholesterol ≤5 mmol/L (OR 1.01; 95% confidence interval [CI] = 0.99 to 1.03; *P* = 0.22) (Table 2).

**Table 2.** Associations of Group 1 outcomes — those dependent on medication adherence with dispensing status

**Code**	**Indicator**	**Dispensing**	**Not dispensing**	**Unadjusted**		**Adjusted**	
		**Median % (IQR)**	**Median % (IQR)**	**OR (CI)**	***P-*value**	**OR (CI)**	***P-*value**
**CHD002**	Percentage of patients with coronary heart disease whose last blood pressure is ≤150/90 mmHg	90.5 (87.6–92.7)	90.1 (86.4–92.8)	1.08 (1.04 to 1.12)	<0.001	1.10 (1.05 to 1.15)	<0.001
**HYP006**	Percentage of patients with hypertension whose last blood pressure is ≤150/90 mmHg	82.2 (78.6–85.2)	80.5 (76.9–83.7)	1.12 (1.09 to 1.15)	<0.001	1.07 (1.04 to 1.11)	<0.001
**PAD002**	Percentage of patients with peripheral arterial disease whose last blood pressure is ≤150/90 mmHg	88.2 (83.6–92.0)	88.2 (82.5–92.7)	1.06 (1.02 to 1.11)	0.008	1.11 (1.05 to 1.17)	<0.001
**STIA003**	Percentage of patients with a history of stroke or transient ischaemic attack whose last blood pressure is ≤150/90 mmHg	85.8 (82.3–89.1)	85.1 (80.8–88.9)	1.07 (1.04 to 1.10)	<0.001	1.07 (1.03 to 1.11)	0.001
**DM002**	Percentage of patients with diabetes whose last blood pressure is ≤150/90 mmHg	88.7 (85.6–91.6)	87.6 (83.8–90.8)	1.12 (1.08 to 1.15)	<0.001	1.10 (1.06 to 1.15)	<0.001
**DM004**	Percentage of patients with diabetes whose last total cholesterol is ≤5 mmol/L	70.3 (66.1–73.6)	69.9 (65.6–73.9)	1.01 (0.99 to 1.03)	0.22	1.03 (1.00 to 1.06)	0.035
**DM007**	Percentage of patients with diabetes whose last HbA1c is ≤59 mmol/mol	64.3 (60.2–68.4)	61.6 (57.1–66.1)	1.12 (1.10 to 1.14)	<0.001	1.01 (0.98 to 1.03)	0.57
**DM008**	Percentage of patients with diabetes whose last HbA1c is ≤64 mmol/mol	72.8 (69.0–76.3)	69.5 (65.0–73.6)	1.18 (1.15 to 1.20)	<0.001	1.02 (0.99 to 1.04)	0.22
**DM009**	Percentage of patients with diabetes whose last HbA1c is ≤75 mmol/mol	84.1 (80.9–86.6)	80.3 (76.2–83.8)	1.28 (1.25 to 1.31)	<0.001	1.04 (1.01 to 1.08)	0.003

CI = confidence interval. HbA1c = haemoglobin A1c. IQR = interquartile range. OR = odds ratio.

**Figure 1.**
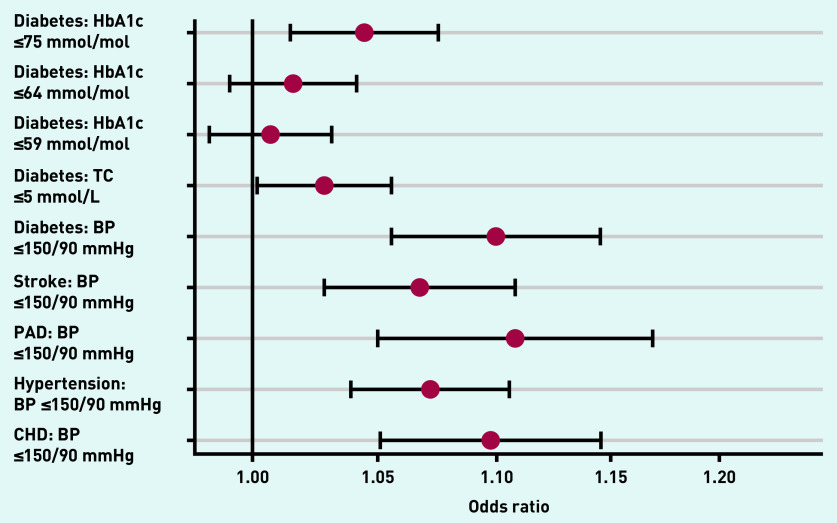
**Differences between dispensing and non-dispensing practices in outcomes dependent on adherence to medication.** **BP = blood pressure. CHD = coronary heart disease. HbA1c = haemoglobin A1c. PAD = peripheral arterial disease. TC = total cholesterol.**

After adjustment there were minor changes in ORs for most indicators. Substantial attenuation of differences in achievement for the three indicators related to haemoglobin A1c (HbA1c) levels was observed. Two of these had *P-*values >0.05: percentage of patients with diabetes whose last HbA1c was ≤59 mmol/mol (OR 1.01; 95% CI = 0.98 to 1.03; *P* = 0.57) and ≤64 mmol/mol (OR 1.02; 95% CI = 0.99 to 1.04; *P* = 0.22). For the remaining seven indicators, achievement was greater for dispensing practices than non-dispensing practices. These included blood pressure targets in hypertension, coronary heart disease, peripheral arterial disease, cerebrovascular disease, and diabetes; achievement of diabetes targets for cholesterol lowering; and for the highest threshold (≤75 mmol/mol) for HbA1c (Table 2, Figure 1).

### Group 2: prescribing indicators independent of adherence

**Figure 2.**
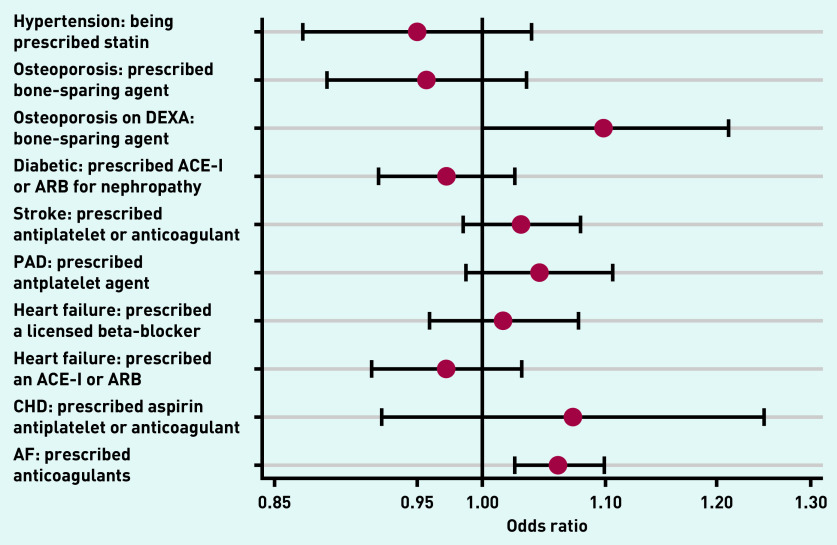
**Differences between dispensing and non-dispensing practices in outcomes dependent on prescription but not adherence.** **ACE-I = angiotensin-converting enzyme inhibitor. AF = atrial fibrillation. ARB = angiotensin receptor blocker. CHD = coronary heart disease. DEXA = dual energy X-ray absorptiometry. PAD = peripheral arterial disease.**

In contrast to Group 1, unadjusted ORs for the 10 prescribing indicators independent of adherence showed no consistency in direction. Only two indicators had ORs >1, with half of the differences showing lower achievement (*P*<0.05). After adjustment the range of ORs was narrower and largely non-discriminatory. Only one statistically significant difference was observed between dispensing and non-dispensing practice: the percentage of patients with atrial fibrillation being prescribed anti-coagulants (OR 1.06; 95% CI = 1.03 to 1.10; *P*<0.001) (Figure 2, Table 3).

**Table 3.** Associations of Group 2 outcomes — those independent of medication adherence with dispensing status

	**Indicator**	**Dispensing**	**Not dispensing**	**Unadjusted**		**Adjusted**	
		**Median % (IQR)**	**Median % (IQR)**	**OR (CI)**	***P-*value**	**OR (CI)**	***P-*value**
**AF007**	Percentage of patients with atrial fibrillation and CHA_2_DS_2_-VASc score ≥2 treated with anticoagulants	82.7 (79.0–86.4)	81.2 (76.2–85.7)	1.12 (1.09 to 1.15)	<0.001	1.06 (1.03 to 1.10)	<0.001
**CHD005**	Percentage of patients with coronary heart disease prescribed antiplatelet agent or an anticoagulant	92.5 (90.2–94.4)	92.7 (90.0–94.8)	1.01 (0.97 to 1.04)	0.75	1.07 (0.92 to 1.24)	0.36
**HF003**	Percentage of patients with left ventricular systolic dysfunction prescribed an ACE-I or ARB	84.8 (77.8–91.7)	86.2 (78.3–100)	0.95 (0.90 to 0.99)	0.017	0.97 (0.92 to 1.03)	0.34
**HF004**	Percentage of patients with left ventricular systolic dysfunction, treated with an ACE-I or ARB, also being prescribed a beta-blocker licensed for heart failure	80.0 (70.0–88.9)	81.8 (72.7–93.6)	0.91 (0.86 to 0.95)	<0.001	1.02 (0.96 to 1.08)	0.58
**PAD004**	Percentage of patients with peripheral arterial disease prescribed aspirin or other antiplatelet agent	88.5 (83.7–92.3)	88.9 (83.3–93.6)	0.92 (0.82 to 1.02)	0.11	1.04 (0.99 to 1.11)	0.14
**STIA007**	Percentage of patients with non-haemorrhagic stroke or TIA prescribed antiplatelet agent or oral anticoagulant	92.5 (90.0–94.7)	92.7 (89.4–95.6)	1.00 (0.97 to 1.04)	0.87	1.03 (0.98 to 1.08)	0.24
**DM006**	Percentage of patients with diabetic nephropathy or micro-albuminuria, prescribed an ACE-I or ARB	80.8 (74.4,87.5)	82.1 (75.0–88.9)	0.92 (0.88 to 0.96)	<0.001	0.97 (0.92 to 1.03)	0.29
**OST002**	Percentage of patients with previous fragility fracture, and osteoporosis on DEXA scanning, prescribed a bone-sparing agent	85.7 (66.7–100)	100 (66.7–100)	0.93 (0.86 to 1.01)	0.080	1.10 (1.00 to 1.21)	0.053
**OST005**	Percentage of patients with previous fragility fracture and osteoporosis prescribed a bone-sparing agent	66.7 (54.6–89.7)	75.0 (57.1–100)	0.88 (0.82 to 0.93)	<0.001	0.96 (0.88 to 1.03)	0.26
**CVD-PP001**	Percentage of patients newly diagnosed with hypertension, with QRISK2 score ≥20%, prescribed a statin	62.5 (50.0–83.3)	75.0 (50.0–100)	0.68 (0.63 to 0.73)	<0.001	0.95 (0.87 to 1.04)	0.26

ACE-I = angiotensin-converting enzyme inhibitor. ARB = angiotensin receptor blocker. CI = confidence interval. DEXA = dual energy X-ray absorptiometry. IQR = interquartile range. OR = odds ratio. TIA = transient ischaemic attack.

### Group 3: prescribing indicators unrelated to medication

There were 27 further QOF indicators not included in the above analyses. ORs in the adjusted analyses showed an overall trend towards higher achievement by dispensing practices (only three ORs being <1); ORs were significantly >1 for 13 (48%) indicators and <1 for none (see Supplementary Table S1 for details).

## DISCUSSION

### Summary

To the authors’ knowledge, this is the first study to consider the impact of primary care dispensing status on differential achievement of QOF indicators for chronic conditions. Evidence for greater achievement by dispensing practices for seven of the nine QOF indicators that depend on adherence to medications was found. In contrast, a difference according to dispensing status was only observed in one of 10 indicators dependent on prescribing but not adherence. Where indicator achievements were unrelated to prescribing, almost half of them were better achieved in dispensing practices.

### Strengths and limitations

This large study analysed data covering >7000 practices in England. These findings are directly relevant to other UK health services, as well as to other countries where access to medications is co-located with primary healthcare settings. The full set of current QOF clinical indicators in unadjusted and adjusted models were examined. The impact of exception reporting on net achievement of QOF indicators has been previously observed by the present authors; therefore, only raw achievement rates were analysed to avoid potential bias due to differences in exception reporting.^9^^,^^28^

This practice-level observational analysis of routine data did not include any direct measures of individual medication adherence, only intermediate outcomes known to depend on good adherence. Medication adherence is affected by individual as well as organisational factors and the authors cannot be sure that their findings reflect impacts on individuals. The observed trends towards greater achievement in dispensing practices of QOF indicators unrelated to prescribing (Group 3) suggest that other organisational characteristics of dispensing practices such as continuity of care, which could not be adjusted for in these analyses, may also be important.^29^ Residual confounding because of this, and other unknown and/or unadjusted factors, is highly likely to be implicated in the findings.^30^ Therefore, the authors do not interpret these findings as clear evidence of differences in medication adherence rates according to practice dispensing status. The results are, however, consistent with the hypothesis that leaving a consultation with a medication, rather than with a prescription that may or may not be dispensed, removes one barrier to medication possession and therefore may plausibly affect medication adherence.

### Comparison with existing literature

Practice characteristics previously associated with greater achievement of QOF indicators in Scotland have included higher deprivation levels, lower income from non-NHS sources, younger ages of GPs, and larger sizes of practice teams.^31^ The rural workforce tends to be older;^32^ lower rates of deprivation and single-handed status were found among dispensing practices in the current study; therefore, lower rather than higher underlying achievement of QOF indicators might have been predicted in dispensing practices. No trend in either direction, however, was evident from the present study control indicators, while adherence indicators uniformly showed higher achievement with dispensing. Evidence relating deprivation to QOF achievement is mixed: associations are weak in magnitude and complex in nature when other barriers to access for the most disadvantaged are accounted for.^28^^,^^33^^,^^34^ It has also been observed that generic indices of deprivation cannot reflect true levels of deprivation in rural areas because of wide heterogeneity of deprivation within such settings.^35^

These findings cannot readily be explained by any systematic differences in quality of care between dispensing and non-dispensing practices. However, the trend to higher achievement of indicators unrelated to prescribing suggests that there may be underlying characteristics of dispensing practices, their patients, or both, contributing to these complex outcomes. Remoteness from urban centres, strongly correlated with dispensing status, does not correlate to a range of measures of quality of care.^36^ Historically, dispensing practice has been associated with lower generic prescribing rates and higher drug unit costs than non-dispensing practice.^37^^,^^38^ No evidence was found for higher rates of prescribing as such in association with dispensing status, thus the ‘perverse incentive’ (now largely mitigated against anyway within the current GP contract) does not account for these findings either. It follows from the hypothesis that dispensing practice drug costs overall will appear to be higher owing to improved medication collection alone, in comparison with non-dispensing practices. In fact, by demonstrating greater achievement of targets for intermediate outcomes such as blood pressure, fewer cardiovascular events and deaths might be predicted. Therefore, to consider drug costs of dispensing practices in isolation, without health economic assessment inclusive of outcomes, is potentially misleading.^38^^,^^39^

Estimates of proportions of prescriptions issued but not dispensed vary widely; the median rate is around 15%.^11^^,^^40^ On-site provision of medication is a distinguishing feature of dispensing practices. Co-location of pharmacies within care settings can improve medication uptake and adherence,^13^ and logistical barriers to medication possession are lower where prescriptions can be dispensed on site or within easy geographical proximity.^14^

### Implications for research and practice

Although barriers to integration of community pharmacy services with primary care exist,^41^ pharmacist engagement in primary care is rising, with roles beyond medication advice increasingly including elements of direct patient care.^9^^,^^42^ Pharmacist-led care can improve medication adherence in long-term conditions such as hypertension,^43^^,^^44^ and such interventions have been shown to save costs and time for GPs.^45^^,^^46^ Community pharmacies are being increasingly co-located with, and/or managed by, primary care teams. Such proximity should facilitate medication adherence. This trend might lead to erosion, in time, of the differences in QOF achievement that have been observed here. The impact of financial incentives on achievement of these quality indicators is also important and may confound time-dependent trends in differences in medication adherence.^47^ No evidence has been found addressing the impact of expanding numbers of pharmacies co-located with surgeries on outcomes such as adherence. Further research on this topic could provide new insights into the importance of ready access to medications, irrespective of the right to dispense medications.

Dispensing directly to patients removes one barrier to medication possession in comparison with prescribing alone. These findings offer initial evidence that dispensing of drugs may result in better intermediate clinical outcomes, as assessed by a range of QOF indicators, in comparison with prescribing alone. A range of organisational and individual factors, which could not be adjusted for, may well have contributed to these observations. The findings are consistent with the hypothesis that differences may be mediated through improved medication adherence; however, it was not possible to directly measure adherence. Further work is required to clarify the possible underlying mechanisms for and significance of these observations, incorporating adherence measures, and to assess the implications for other models of primary care dispensing such as on-site pharmacies.
